# Improved diagnosis of histological capsule in hepatocallular carcinoma by using nonenhancing capsule appearance in addition to enhancing capsule appearance in gadoxetic acid-enhanced MRI

**DOI:** 10.1038/s41598-023-33048-8

**Published:** 2023-04-14

**Authors:** Eiko Nishioka, Keitaro Sofue, Koji Maruyama, Eisuke Ueshima, Yoshiko Ueno, Masakatsu Tsurusaki, Shohei Komatsu, Takumi Fukumoto, Takamichi Murakami

**Affiliations:** 1 Department of Radiology, Kobe University Graduate School of Medicine, Kobe, Japan; 2 Department of Radiology, Faculty of Medicine, Kindai University, Osaka-Sayama, Japan; 3 Division of Hepato-Biliary-Pancreatic Surgery, Department of Surgery, Kobe University Graduate School of Medicine, Kobe, Japan

**Keywords:** Cancer, Gastroenterology

## Abstract

To assess the value of nonenhancing capsule by adding to enhancing capsule in gadoxetic acid-enhanced MRI (EOB-MRI) in comparison with contrast-enhanced CT (CE-CT) for diagnosing histological capsule in hepatocellular carcinoma (HCC). One-hundred fifty-one patients with HCC who underwent both CE-CT and EOB-MRI were retrospectively reviewed. Liver Imaging-Reporting and Data System (LI-RADS) v2018 imaging features, including enhancing and nonenhancing capsule were evaluated by two readers in CE-CT and EOB-MRI. Frequencies of each imaging feature were compared between CE-CT and EOB-MRI. The area under the receiver operating characteristic (AUC) curve for the diagnosis of histological capsule was compared across the following three imaging criteria: (1) enhancing capsule in CE-CT, (2) enhancing capsule in EOB-MRI, and (3) enhancing/nonenhancing capsule in EOB-MRI. Enhancing capsule in EOB-MRI was significantly less frequently depicted than that in CE-CT (*p* < 0.001 and = 0.016 for reader 1 and 2). Enhancing/nonenhancing capsule in EOB-MRI achieved a similar frequency of enhancing in CE-CT (*p* = 0.590 and 0.465 for reader 1 and 2). Adding nonenhancing capsule to enhancing capsule in EOB-MRI significantly increased AUCs (*p* < 0.001 for both readers) and achieved similar AUCs compared with enhancing capsule in CE-CT (*p* = 0.470 and 0.666 for reader 1 and 2). Adding nonenhancing capsule to the definition of capsule appearance can improve the diagnosis of capsule in EOB-MRI for the diagnosis of histological capsule in HCC and decrease discordance of capsule appearance between EOB-MRI and CE-CT.

## Introduction

Hepatocellular carcinoma (HCC) is the fifth common malignancy in the world, and its incidence is increasing in the United States^1,2^. Computed tomography (CT) and magnetic resonance imaging (MRI) play a pivotal role in the noninvasive diagnosis and staging of HCC, as early detection and treatment lead to improved prognosis and outcomes^3^. Several imaging diagnostic systems have been developed and introduced for the diagnosis of HCC, and arterial phase hyperenhancement (APHE) followed by washout appearance are the hallmark imaging features^4–7^.

The Liver Imaging-Reporting and Data System (LI-RADS) is an imaging-based diagnostic algorithm that standardizes the diagnosis of HCC in patients at risk for HCC^8,9^. Gadoxetic acid is one of the hepatobiliary MR contrast agents, and gadoxetic acid-enhanced MRI (EOB-MRI) offers dynamic phase imaging followed by hepatocyte-specific imaging during the hepatobiliary phase (HBP). The primary advantages of EOB-MRI are derived from higher detection sensitivity and differentiation of HCCs from well-differentiated benign or low-grade hepatocellular lesions and hypervascular pseudolesions^10–14^. EOB-MRI has several disadvantages, including difficulty in obtaining optimal arterial phase and separating vascular washout from hepatobiliary uptake of the agent after of the portal venous phase^15–17^.

An enhancing capsule is one of the major imaging features in the LI-RADS diagnostic algorithm. This conventional capsule appearance is defined as a peripheral rim of smooth hyperenhancement that surrounds an observation. A histological fibrous capsule retains extracellular contrast agents longer than surrounding liver parenchyma, especially on equilibrium phase images^18,19^. On EOB-MRI, however, rapid clearance from the blood pool and simultaneous hepatocyte uptake of the contrast agent precludes obtaining the equilibrium state, resulting in lower depiction of the enhancing capsule compared with extracellular contrast agents^20–23^. However, several works have demonstrated that the finding of a smooth hypointense rim on HBP images can be used to detect a histological capsule in EOB-MRI^24,25^. We hypothesize that adding nonenhancing capsule appearance (i.e. HBP hypointense rim) to the definition of capsule appearance could improve the diagnostic performance of EOB-MRI for the detection of histological capsule in HCCs.

The purpose of this study was to assess the value of nonenhancing capsule by adding to enhancing capsule in EOB-MRI in comparison with CE-CT for diagnosing histological capsule in HCC.

## Methods

This study protocol conforms to the Declaration of Helsinki and Ethical Guidelines for Medical and Health Research Involving Human Subjects in Japan. This retrospective study was approved by the Kobe University Ethical Committee (Permission number: B200008) and carried out according to the guidelines of the committee. The Kobe University Ethical Committee waived the need for an informed consent.

### Patients

A search of our institution’s surgical database from July 2011 and December 2016 identified 275 consecutive patients who underwent surgical resection for pathologically confirmed HCCs. One abdominal radiologist (E.U.) and one surgeon (S.K.) reviewed the surgical and radiological database and identified 177 patients who met the following criteria: (1) patients at high risk for HCC according to LI-RADS v2018, (2) patients who had a pathologically confirmed single to three HCCs, and (3) patients who underwent both CE-CT and EOB-MRI no more than 2 months before the resection of HCC. After reviewing the imaging examination and pathological reports, 26 patients were further excluded for one of the following reasons: (i) patients had undergone a preoperative treatment for HCC (n = 6) or (ii) imaging examination and pathological records revealed macrovascular invasion of HCC (n = 20). Finally, 151 patients who had single to three HCCs were included in this study.

The study population consisted of 151 patients (mean age, 68.9 ± 9.6 years; range, 37–85 years), including 124 men (mean age, 68.1 ± 9.8 years; range, 37–85 years) and 27 women (mean age, 72.1 ± 8.1 years; range, 46–85 years). This study delt with only one dominant HCC for image analysis, because the presence of a histological capsule was recorded for the most dominant HCC in the pathological reports. Detailed patient demographic information was obtained from the electronic medical records and are summarized in Table 1.

**Table 1:** Baseline characteristics of the study population.

Variable	Value
Patient characteristics	
Age (year)	
Mean ± SD (range)	68.9 ± 9.6 (37–85)
Gender	
Male	124 (82.1)
Female	27 (17.9)
Underlying liver disease	
Hepatitis C virus	61 (40.4)
Hepatitis B virus	26 (17.2)
Hepatitis B and C virus	3 (2.0)
Nonalcoholic steatohepatitis	43 (28.5)
Alcoholic	14 (9.3)
Others	4 (2.6)
Child–Pugh classification	
A	149 (98.7)
B	2 (1.3)
C	0 (0)
AFP (ng/mL)	
Median (interquartile range)	11 (5–53)
< 20 ng/mL	92 (60.9)
≥ 20 ng/mL	59 (39.1)
Histopathological findings	
Tumor size of HCC (mm)	
Median (interquartile range)	38 (24–62)
< 10 mm	4 (2.7)
10–20 mm	28 (18.5)
≥ 20 mm	119 (78.8)
Fibrous capsule in HCC	
Presence	124 (82.1)
Absence	27 (17.9)
Histological differentiation of HCC	
Well-differentiated	14 (9.3)
Moderately-differentiated	115 (76.2)
Poorly-differentiated	22 (14.5)
METAVIR fibrous score	
F0	15 (9.9)
F1	26 (17.2)
F2	47 (31.1)
F3	39 (25.9)
F4	24 (15.9)

Data are expressed as count (percentage) for categorical variables.

*AFP* α-fetoprotein, *HCC* hepatocellular carcinoma.

### MR Image acquisition

All MR examinations were performed on either 1.5 T (n = 43, Achieva 1.5, Philips Medical Systems) or 3 T (n = 108, Achieva 3.0 T and Ingenia 3.0 T, Philips Medical Systems; Vantage Tital 3 T, Canon Medical Systems) clinical MR systems. The baseline MRI examinations included a single-shot turbo spin-echo, breath-hold gradient dual-echo T1-weighted (in-phase and opposed-phase), fat-suppressed fast spin-echo T2-weighted, diffusion-weighted (b = 0, 500, and 1000 s/mm^2^, applied in three orthogonal directions), and dynamic contrast-enhanced sequences. The dynamic contrast-enhanced sequences were obtained using a 3D fat-suppressed T1-weighted spoiled gradient-recalled echo pulse sequence. After obtaining precontrast images, intravenous gadoxetic acid (Primovist, Bayer Pharma) was administered at a dose of 0.025 mmoL/kg body weight at a rate of 2.0 mL/sec, followed by a 20-mL saline flush at 1.0–2.0 mL/sec. Postcontrast images were obtained during the arterial phase (27–40 s using fluoroscopic triggering technique), portal venous phase (70 s), and transitional phase (120 s) images after an intravenous administration of gadoxetic acid. HBP images were obtained 20 min after the administration of contrast agent.

### CT Image acquisition

CE-CT examinations were performed using either 64-detector row CT systems (Aquillion 64 or One; Cannon Medical Systems). Scan parameters were: detector configuration, 64 × 0.5 or 80 × 0.5 mm; acquisition matrix, 512 × 512; gantry rotation, 0.5 s; pitch factor, 0.641; tube voltage, 120 kVp; tube current–time product, automatic exposure control. All CT image data were reconstructed in the axial plane with a 5-mm thickness and 5-mm reconstruction interval. Iodinated nonionic contrast material was injected into an antecubital vein using an automatic power injector at a dose of 600 mg I/kg of patient body weight, up to maximum dose of 47.25 g of iodine. The injection duration was fixed at 25 s, and the injection rate varied (2.4–4.3 mL/sec.) depending on the patients’ body weight. Precontrast images were initially acquired, and after intravenous administration of the contrast material, the arterial phase (33–40 s using bolus tracking technique), portal venous phase (70 s), and equilibrium phase (150–180 s) images were acquired. Bolus-tracking technique was used to obtain hepatic arterial phase images 12 s after the trigger threshold was achieved. All CT data were reconstructed in the axial plane with 5-mm section thickness and 5-mm reconstruction interval by using a standard soft tissue kernel.

### Image analysis

Image analyses were independently performed by two abdominal radiologists (K.M. and K.S.) who were aware that the patients had pathologically confirmed HCCs but were blinded to all other information, including clinical history, imaging findings, and pathological findings. A study coordinator reviewed pathology reports in which the presence of a histological capsule was described for the most dominant HCC and presented the location of the HCC in each imaging examination to each reader since our institution’s pathology reports precisely describe only the single dominant HCC. To minimize the effects of recall bias, two image analysis sessions separated by an 8-weeks interval were applied since readers reviewed both CT and MRI images and evaluated similar imaging features for the same patients. In the first session, each reviewer assessed half of the MR and half of the CT examinations in random order, and then assessed the other examination for each patient in random order in the second session.

**Figure 1:**
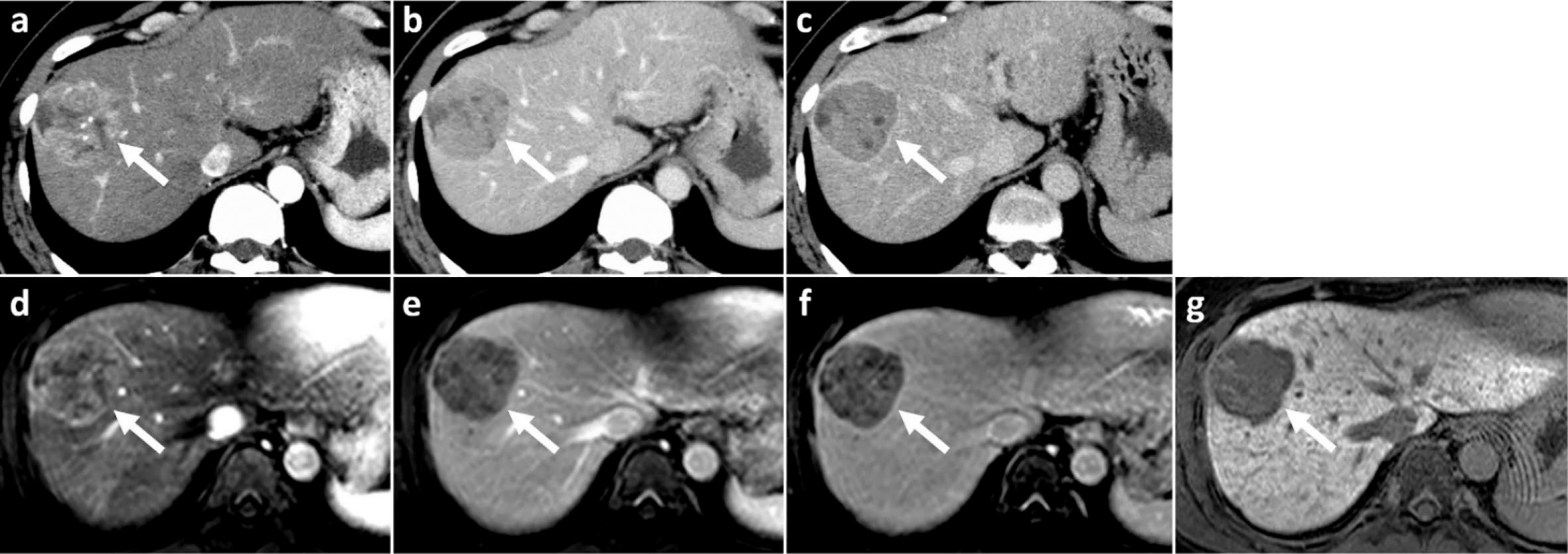
CT and MR images in a 37-year-old male with poorly-differentiated HCC with histological capsule. METAVIR score of the background liver was F3. In contrast-enhanced CT, a 49-mm observation shows (**a**) nonrim APHE on arterial phase and (**b**) nonperipheral washout with an enhancing capsule on equilibrium phase. In gadoxetic acid-enhanced MRI, a 48-mm observation shows (**c**) nonrim APHE on arterial phase, (**d**) nonperipheral washout on portal venous phase, and (**e**) TP hypointensity with suspicion of an enhancing capsule on transitional phase. Hepatobiliary phase image clearly shows (**f**) a nonenhancing capsule surrounding the observation.

CT/MRI LI-RADS v2018 terminology and definitions were used for the assessment of the imaging features of each nodule^7^. First, each reader measured the size of each lesion in the axial plane on the dynamic phase images in which the nodule’s margins were most sharply demarcated. Next, LI-RADS major features (nonrim arterial phase hyperenhancement [nonrim APHE], nonperipheral washout, enhancing capsule) were determined based on dynamic features only. Diagnostic confidence for the presence of each feature was assigned using the following five-point Likert scale: 1, definitely absent; 2, probably absent; 3, equivocal; 4, probably present; and 5, definitely present. Threshold growth was not assessed because prior imaging examinations were not provided in this study. Nonrim APHE, nonperipheral washout, and enhancing capsule were assessed using the LI-RADS v2018 definitions. In addition to the LI-RADS v2018 definition of enhancing capsule, the presence of nonenhancing capsule, defined as capsule appearance not visible as an enhancing rim including smooth peripheral hypointense rim on the HBP image^24^, was also recorded. The finding of nonenhancing capsule appearance is considered an ancillary feature favoring malignancy in LI-RADS v2018. When a confidence level of 4 or 5 was assigned to a feature, it was considered to be present (Fig. 1).

### Histopathologic analysis

The presence or absence of a histopathological fibrous capsule is described in postoperative pathologic reports at our institution in cases of HCC; this description was used as the reference standard for the presence of a capsule. All pathologic assessments were performed by a team of pathologists, each one with more than 10 years of experience in liver pathology. Notably, this description is only provided for the dominant HCC in our pathology reports. A capsule was considered present when a fibrous capsule was visualized along at least one-third of the tumor margin, regardless of the presence of a microscopic capsular or extracapsular invasion. The tumor size, histopathologic features of the presence of capsule, degree of tumor differentiation, and fibrosis score of background liver parenchyma were recorded. Tumor differentiation was classified based on the World Health Organization classification system^26^, and liver fibrosis was graded based on the METAVIR fibrosis scoring system^27^.

### Statistical analysis

Continuous variables were summarized as mean ± standard deviation or median and interquartile range, where appropriate. Categorical variables were presented as counts and frequencies. Interobserver agreement for LI-RADS imaging features was assessed by using the intraclass correlation coefficient (ICC) for continuous variables and the weighted kappa coefficient (κ) for categorical variables. They were interpreted as follows: poor, 0.00–0.20; fair, 0.20–0.40; moderate, 0.40–0.60; substantial, 0.60–0.80; and almost perfect agreement, 0.80–1.00.

The frequencies of each LI-RADS imaging feature were compared between CE-CT and EOB-MRI using the Wilcoxon’s signed rank test and McNemar’s test. Additionally, frequencies between the enhancing capsule in CE-CT and enhancing/nonenhancing capsule were also compared using the McNemar test. To assess diagnostic performance for the presence of histological capsule, sensitivity, specificity, positive predictive value (PPV), negative predictive value (NPV), and accuracy were calculated along with their 95% confidence intervals and compared using the Fisher’s exact test between the following three imaging features: (1) enhancing capsule in CE-CT, (2) enhancing capsule in EOB-MRI, and (3) enhancing/nonenhancing capsule in EOB-MRI. The area under the receiver operating characteristic curve was also calculated and compared between the three imaging features.

For all statistical analyses, two-tailed *P* values under 0.05 indicated statistical significance. Statistical analyses were performed using commercially available software (SPSS version 20.0; IBM, Chicago, IL).

## Results

### Histopathological characteristics of HCC

Patient characteristics and histologic findings are presented in Table 1. A histopathological examination showed that the median tumor size was 38 mm. One hundred twenty-seven (82.1%) HCCs had either a complete (n = 35) or partial (n = 92) fibrous capsule, and 33 (23.8%) HCCs were found to have microvascular invasion on pathological examination. Most (76.2%) tumors were histologically diagnosed as well-differentiated HCCs. The background liver fibrosis was various based on the METAVIR fibrous score.

### LI-RADS imaging features in CE-CT and EOB-MRI.

Interobserver agreement assessments for LI-RADS imaging features are summarized in Table 2. Interobserver agreement was almost perfect for observation size (ICC = 0.987and 0.982 for CE-CT and EOB-MRI) and substantial for APHE (κ = 0.690 and 0.681 for CE-CT and EOB-MRI), Washout (κ = 0.614 and 0.692 for CE-CT and EOB-MRI), and transitional phase hypointensity (κ = 0.605 for EOB-MRI). With regard to the enhancing capsule, interobserver agreement was substantial in CE-CT (κ = 0.689) but only moderate in EOB-MRI (κ = 0.597). Although statistical comparisons could not be applied, interobserver agreement slightly increased when the nonenhancing capsule was added to the enhancing capsule in EOB-MRI (κ = 0.667).

**Table 2:** Interobserver Agreement for LI-RADS Imaging Features of HCCs in Contrast-Enhanced CT and Gadoxetic Acid-Enhanced MRI.

Variable	CE-CT	EOB-MRI
Observation size (mm)	0.987 (0.984–0.990)	0.982 (0.977–0.987)
Nonrim APHE	0.690 (0.421–0.959)	0.681 (0505–0.872)
Nonperipheral washout	0.614 (0.490–0.739)	0.692 (0.613–0.771)
TP hypointensity	–	0.605 (0.492–0.719)
Enhancing capsule	0.689 (0.615–0.765)	0.597 (0.508–0.687)
Enhancing/nonenhancing capsule	–	0.667 (0.581–0.752)

Data are expressed as intraclass correlation coefficient (ICC) for observation size and as κ value for nonrim APHE, nonperipheral “washout”, TP hypointensity, enhancing “capsule”, and nonenhancing/enhancing “capsule”.

Data in parentheses are expressed as 95% confident intervals.

*CE-CT* contrast-enhanced CT, *EOB-MRI* gadoxetic acid-enhanced MRI, *APHE* arterial phase hyperenhancement, *TP* transitional phase.

**Figure 2:**
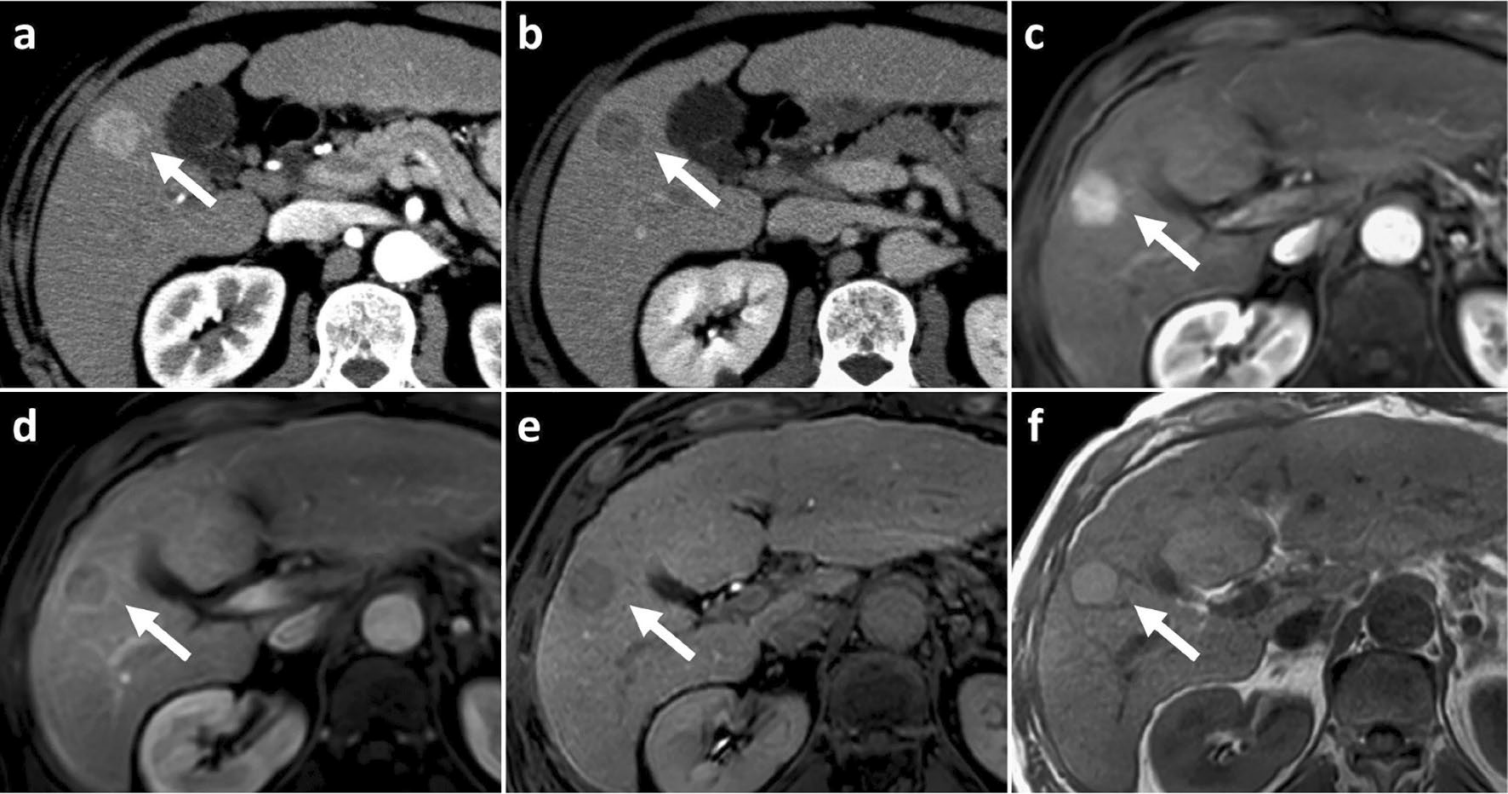
CT and MR images in a 71-year-old male with moderately-differentiated HCC with histological capsule. METAVIR score of the background liver was F4. In contrast-enhanced CT, an 18-mm observation shows (**a**) nonrim APHE on arterial phase and (**b**) nonperipheral washout with an ambiguous enhancing capsule on equilibrium phase. In gadoxetic acid-enhanced MRI, a 19-mm observation shows (**c**) nonrim APHE on arterial phase and (**d**) TP hypointensity and enhancing capsule on transitional phase. Although (**e**) the presence of a nonenhancing capsule is obsecure on hepatobiliary phase, (**f**) T1-weighted image clearly shows a nonenhancing capsule surrounding the observation.

The comparison of the frequencies of LI-RADS imaging features is summarized in Table 3. Between the two readers, no significant difference was detected in observation size between in CE-CT and EOB-MRI (*p* = 0.082 and 0.118 for reader 1 and 2). Although most HCCs were designated as having nonrim APHE both in CE-CT (94.7% and 93.4% for reader 1 and 2) and EOB-MRI (90.7% and 87.4% for reader 1 and 2), the difference in frequency was statistically significant for both readers (*p* = 0.068 and 0.012 for reader 1 and 2). Nonperipheral washout was significantly less frequently observed in EOB-MRI (70.9% and 74.8% for reader 1 and 2) than in CE-CT (88.7% and 87.4% for reader 1 and 2) (*p* < 0.001 for both readers). Enhancing capsule in EOB-MRI (56.3% and 61.6% for reader 1 and 2) was significantly less frequently visualized than enhancing capsule in CE-CT (71.5% and 70.9% for reader 1 and 2) (*p* < 0.001 and = 0.016 for reader 1 and 2). The combined definition of enhancing/nonenhancing capsule achieved a similar detection frequency (74.2% and 72.8% for reader 1 and 2) of enhancing capsule in CE-CT (*p* = 0.590 and 0.465 for reader 1 and 2) (Fig. 2).

**Table 3:** Frequencies of LI-RADS Imaging Features of HCCs between in Contrast-Enhanced CT and Gadoxetic Acid-Enhanced MRI.

Variable	Reader 1			Reader 2		
	CE-CT	EOB-MRI	*p* value	CE-CT	EOB-MRI	*p* value
Observation size (mm)	48.1 ± 34.8	47.6 ± 34.8	.082	47.4 ± 34.2	47.1 ± 35.2	.118
Nonrim APHE	94.7% (143/151)	90.7% (137/151)	.068	93.4% (141/151)	87.4% (132/151)	.012
Non peripheral washout	88.7% (134/151)	70.9% (107/151)	< .001	87.4% (132/151)	74.8% (113/151)	< .001
TP hypointensity	–	88.1% (133/151)	.203	–	90.1% (136/151)	.134
Enhancing capsule	71.5% (108/151)	56.3% (85/151)	< .001	70.9% (107/151)	61.6% (93/151)	.016
Enhancing/nonenhancing capsule	–	74.2% (112/151)	.59	–	72.8% (110/151)	.465

Data are expressed as mean ± standard deviation for continuous variable and frequency (count) for categorical variables.

The frequencies of TP hypointensity was compared with nonperipheral “washout”, and enhancing/nonenhancing “capsule” in EOB-MRI was compared with enhancing “capsule” in CE-CT.

*CE-CT* contrast-enhanced CT, *EOB-MRI* gadoxetic acid-enhanced MRI, *APHE* arterial phase hyperenhancement, *TP* transitional phase.

### Diagnostic performance for the presence of histological capsule

**Figure 3:**
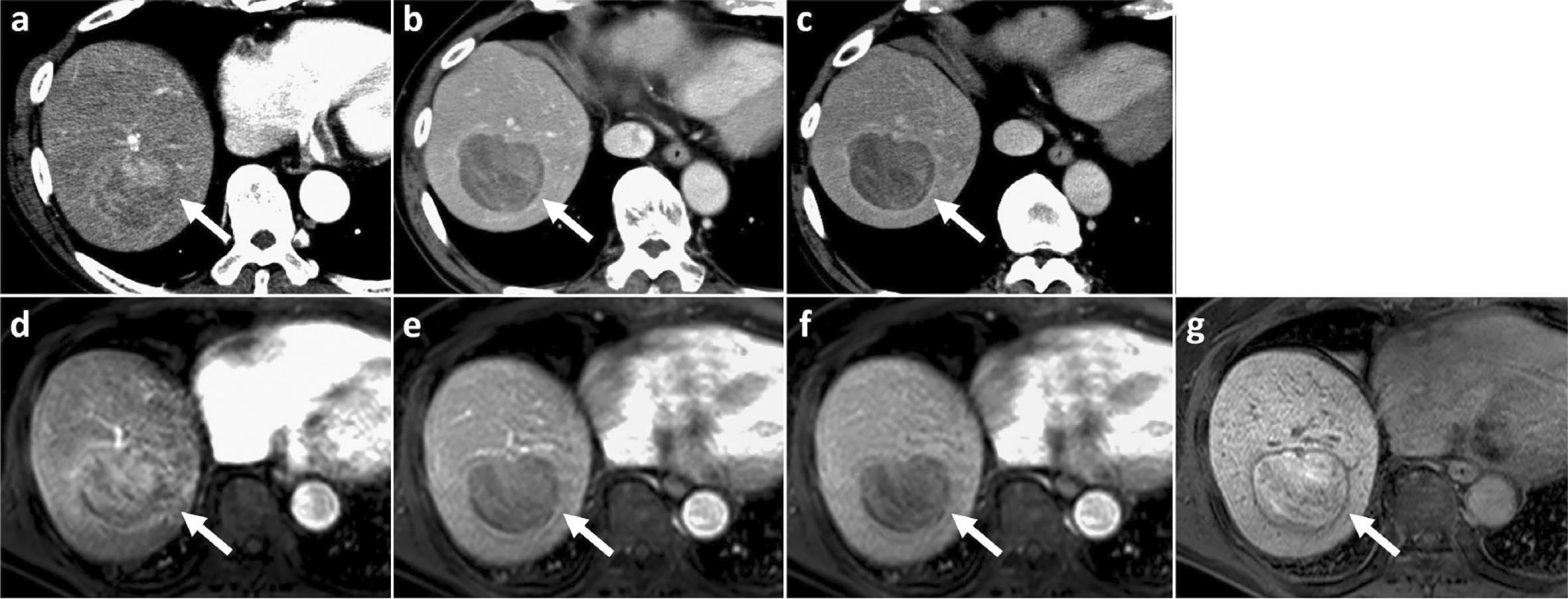
CT and MR images in a 75-year-old male with moderately-differentiated HCC with histological capsule. METAVIR score of the background liver was F1. In contrast-enhanced CT, a 59-mm observation shows (**a**) nonrim APHE on arterial phase and nonperipheral washout with a definite enhancing capsule on (**b**) portal venous and (**c**) equilibrium phase. In gadoxetic acid-enhanced MRI, a 60-mm observation shows (**d**) nonrim APHE on arterial phase, (**e**) washout on portal venous phase and (**f**) TP hypointensity on transitional phase. An enhancing capsule is not definitely present on either portal venous or transitional phase images. On hepatobiliary phase image (**g**), the observation demonstrates iso/hyperintense, and a surrounding smooth hypointense rim is observed as a nonenhancing capsule.

The diagnostic performance of the three imaging features (enhancing capsule in CE-CT, enhancing capsule in EOB-MRI, and enhancing/nonenhancing capsule in EOB-MRI) for predicting the presence of a histological capsule is summarized in Table 4. The sensitivity and accuracy of enhancing capsule in CE-CT were higher than those of enhancing capsule in EOB-MRI (*p* = 0.002 and 0.035 for reader 1 and 2 regarding sensitivity, and *p* = 0.005 and 0.078 for reader 1 and 2 regarding accuracy), while the specificity (*p* = 1.00 and 0.785 for reader 1 and 2), PPV (*p* = 0.793 and 1.00 for reader 1 and 2), and NPV (*p* = 0.106 and 0.404 for reader 1 and 2) were not significantly different for either reader . After adding the nonenhancing capsule component to the definition of enhancing capsule in EOB-MRI, 27 and 17 observations were changed from false negatives to true positives for observers 1 and 2, respectively (Fig. 3). One observation for reader 1 and no observations for reader 2 were converted from a true negative to a false positive by changing the definition. Consequently, the sensitivity and accuracy of enhancing/nonenhancing capsule in EOB-MRI increased and showed no significant differences in comparison with enhancing capsule in CE-CT (*p* = 0.618 and 0.431 for reader 1 and 2 regarding sensitivity, and *p* = 0.775 and 0.491 for reader 1 and 2 regarding accuracy), while retaining the original specificity (*p* = 0.779 and 0.785 for reader 1 and 2), PPV (*p* = 1.00 and 0.813 for reader 1 and 2), and NPV (*p* = 0.826 and 0.659 for reader 1 and 2). When nonenhancing capsule was considered as a major feature of LI-RADS in addition to enhancing capsule, three LR-4 observations were recategorized as LR-5 for reader 1, and one LR-3 and LR-4 observations were recategorized as LR-4 and LR-5 for reader 2, respectively.

**Table 4:** Diagnostic performance for the Presence of Histological Capsule in HCCs among Three Imaging Features.

Variable	(1) Enhancing capsule in CE-CT	(2) Enhancing capsule in EOB-MRI	(3) Enhancing/nonenhancing capsule in EOB-MRI	*P* value	
				(1) versus (2)	(1) versus (3)
Reader 1					
Sensitivity	80.6% (100/124)	62.1% (77/124)	83.9% (104/124)	.002	.618
	(95% CI 77.1–83.4)	(95% CI 58.4–65.0)	(95% CI 80.3–86.8)		
Specificity	70.4% (19/27)	70.4% (19/27)	66.7% (18/27)	1	.779
	(95% CI 53.9–83.1)	(95% CI 53.4–83.5)	(95% CI 50.4–79.9)		
Positive predictive value	92.6% (100/108)	90.6% (77/85)	92.0% (104/113)	.793	1
	(95% CI 88.5–95.8)	(95% CI 85.2–94.8)	(95% CI 88.1–95.2)		
Negative predictive value	44.2% (19/43)	28.8% (19/66)	47.4% (18/38)	.106	.826
	(95% CI 33.9–52.2)	(95% CI 21.8–34.2)	(95% CI 35.8–56.8)		
Accuracy	77.8% (119/151)	63.6% (96/151)	80.8% (122/151)	.005	.775
	(95% CI 72.9–83.4)	(95% CI 57.5–68.3)	(95% CI 75.0–85.5)		
Reader 2					
Sensitivity	78.2% (97/124)	66.1% (82/124)	82.3% (102/124)	.035	.431
	(95% CI 74.6–81.3)	(95% CI 62.4–69.1)	(95% CI 78.7–85.2)		
Specificity	63.0% (17/27)	66.7% (18/27)	66.7% (18/27)	.785	.785
	(95% CI 46.4–77.1)	(95% CI 49.7–80.5)	(95% CI 50.3–80.0)		
Positive predictive value	90.7% (97/107)	90.1% (82/91)	91.9% (102/111)	1	.813
	(95% CI 86.5–94.2)	(95% CI 85.1–94.2)	(95% CI 87.9–95.1)		
Negative predictive value	38.6% (17/44)	30.0% (18/60)	45.0% (18/40)	.404	.659
	(95% CI 28.5–47.3)	(95% CI 22.4–36.2)	(95% CI 33.9–54.0)		
Accuracy	75.5% (114/151)	66.2% (100/151)	79.5% (120/151)	.078	.491
	(95% CI 69.6–80.6)	(95% CI 60.1–71.2)	(95% CI 73.6–84.2)		

Data are expressed as frequency (count).

*95%CI* 95% confident interval, *CE-CT* contrast-enhanced CT, *EOB-MRI* gadoxetic acid-enhanced MRI.

**Figure 4:**
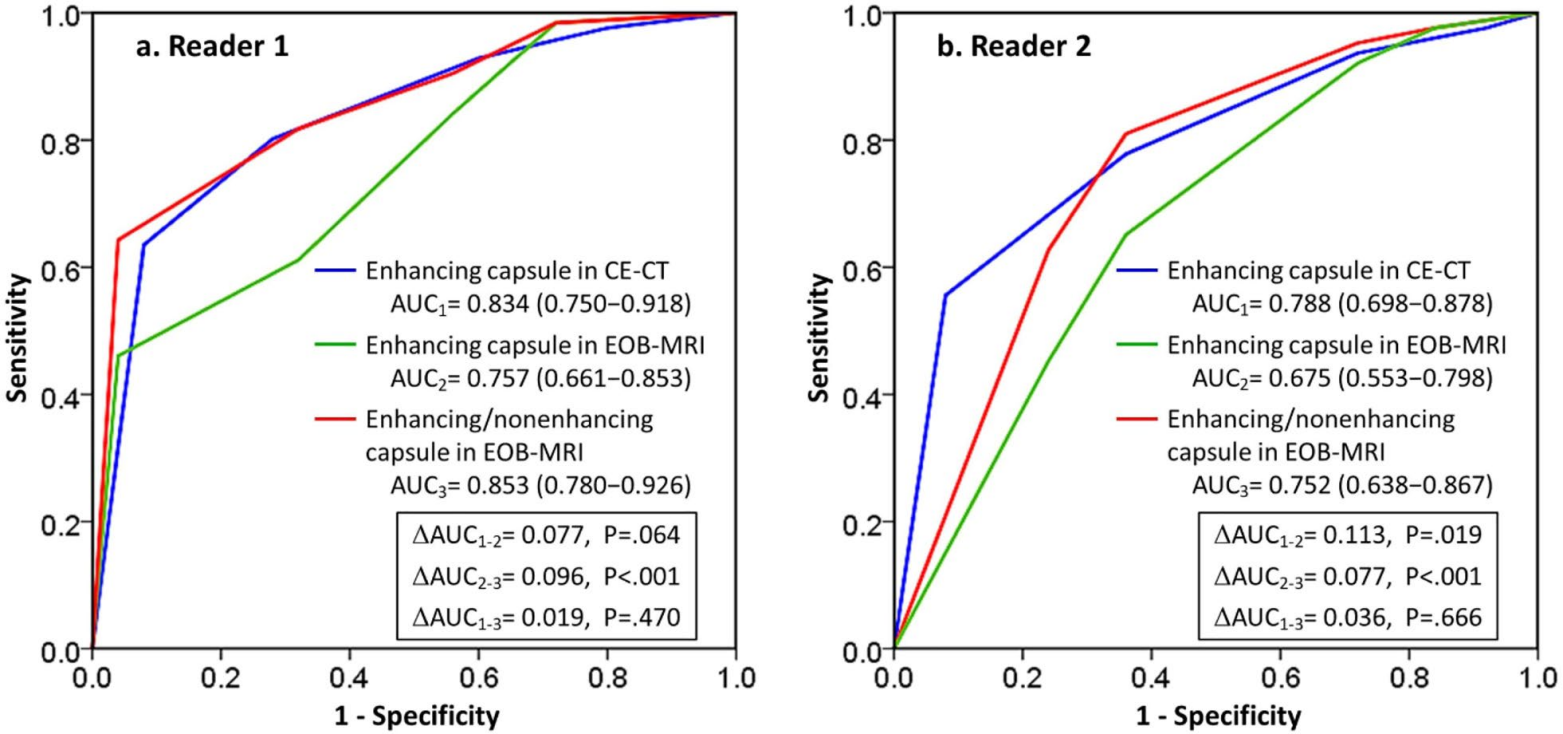
Receiver operating characteristics curves for enhancing capsule in CE-CT (blue), enhancing capsule in EOB-MRI (green), and enhancing/nonenhancing capsule in EOB-MRI (red) for the diagnosis of histological capsule in (**a**) reader 1, and (**b**) reader 2. AUC = area under the receiver operating characteristic curve.

The area under the receiver operating characteristic curve (AUC) for detecting the presence of a histological capsule using CE-CT was higher than that for EOB-MRI both in reader 1 (AUC: 0.834 vs 0.757, *p* = 0.064) and reader 2 (AUC: 0.788 vs 0.675, *p* = 0.019) according to the standard definition of a Capsule (Fig. 4). The modified definition of enhancing/nonenhancing capsule in EOB-MRI significantly increased the AUC for both reader 1 (AUC: 0.853 vs 0.757, *p* < 0.001) and reader 2 (AUC: 0.752 vs 0.675, *p* < 0.001), and achieved a similar diagnostic performance compared with enhancing capsule in CE-CT for both reader 1 (AUC: 0.853 vs 0.834, *p* = 0.470) and reader 2 (AUC: 0.752 vs 0.788, *p* = 0.666).

## Discussion

The present study demonstrated that gadoxetic acid-enhanced MRI reveals enhancing capsule less frequently and has an inferior diagnostic performance for the presence of a histological capsule in hepatocellular carcinoma compared with contrast-enhanced CT. On the other hand, adding nonenhancing capsule to enhancing capsule in gadoxetic acid-enhanced MRI significantly increases the detection frequencies of capsule appearance and diagnostic performance for the presence of a histological capsule in hepatocellular carcinoma, which is comparable with contrast-enhanced CT. Our findings indicate that adding the nonenhancing capsule to the definition of capsule appearance can improve the diagnostic performance of the hepatobiliary contrast agents and decrease discordance between imaging examinations with hepatobiliary and extracellular contrast agents in the Liver Imaging-Reporting and Data System diagnostic algorithm.

Although its value was controversial previously published studies^19,28–30^, the capsule appearance has been highlighted since the LI-RADS criteria incorporate it as a major imaging feature for the diagnosis of HCC. The presence of capsule appearance has also been shown to be beneficial prognostically since it predicts lower recurrence rates after hepatic resection, ablation, and transarterial embolization^31–33^. Our study demonstrated that EOB-MRI revealed lower detection frequencies for enhancing capsule, which is consistent with previous studies^20–24^. On the other hand, adding HBP hypointense rim to the definition of capsule appearance in EOB-MRI significantly increased the diagnostic performance of a histological fibrous capsule, which had a similar accuracy as other imaging modalities with extracellular contrast agents, both in our results and previous papers^18,19,28^. We believe that the finding of nonenhancing capsule can help compensate for the drawback of non-visualized enhancing capsule in EOB-MRI. A HBP hypointense rim may be more conspicuous when the surrounding background liver parenchyma or tumor is more hyperintense, such as when liver function is preserved and for HBP-hyperintense HCC. Meanwhile, a conventional enhancing capsule appearance may be easily detectable even in EOB-MRI when significant fibrosis or severe liver dysfunction exists since hepatic uptake of the contrast agent is delayed, and retention of the contrast agent in the fibrous capsule may be more conspicuous. Other factors, including the size of the HCC, histological differentiation of HCC, acquisition parameters, and timing of the transitional phase and HBP acquisitions, could also influence the visualization of capsule appearance in EOB-MRI, thereby warranting further studies to clarify which factors are significantly associated with the visualization of capsule appearance.

LI-RADS incorporates specific standardized definitions in an attempt to improve reproducibility across institutions, readers, and imaging modalities. Interobserver agreement regarding LI-RADS major imaging features in EOB-MRI has been previously assessed, and substantial disagreement for enhancing capsule has been considered an issue^22,23,34^. An inferior agreement with regard to the finding of enhancing capsule in EOB-MRI compared with that in CE-CT was also observed in our study. Adding nonenhancing capsule to enhancing capsule could increase interobserver agreement for the identification of capsule appearance, which may further improve reproducibility in the context of LI-RADS categorization. Interexamination variability, particularly between CE-CT and EOB-MRI, is also a cause for concern when using LI-RADS and other imaging diagnostic algorithms^22,35^. Our result showing that using nonenhancing capsule could reduce discordance between CE-CT and EOB-MRI was a notable finding for preserving the reproducibility of the LI-RADS diagnostic algorithm.

Our study has a number of limitations. First, it was based on a single-center retrospective design with a histopathological reference standard, and patient selection bias was likely present. However, we included consecutive patients who underwent both CE-CT and EOB-MRI followed by surgical resection to minimize recruitment bias. Second, most HCCs were large (≥ 20 mm) because small HCCs were often treated using locoresional therapies without pathological confirmation at our institution. Third, this study only focused on HCCs and did not include other focal liver observations, including benign focal lesions, borderline hepatic nodules, or other malignant lesions. Further studies are warranted to assess the effect of adding nonenhancing to the definition of capsule appearance on diagnostic accuracy for HCC in EOB-MRI when using the LI-RADS diagnostic algorithm. Last, our study only compared the LI-RADS major imaging features between CE-CT and EOB-MRI. Thus, further studies are needed to determine whether our findings are applicable to the comparison between EOB-MRI and MRI with extracellular contrast agents, in which enhancing capsule are most frequently detected^20,21,23^.

In conclusion, including nonenhancing HBP hypointense rim in addition to enhancing capsule for the definition of capsule appearance can improve the detection frequency and diagnostic accuracy of EOB-MRI for histological capsule in HCC. This also improves interobserver agreement and decreases discordance between gadoxetic acid-enhanced MRI and contrast-enhanced CT when using the Liver Imaging-Reporting and Data System v2018 diagnostic algorithm.

## Data Availability

The datasets used and/or analysed during the current study available from the corresponding author on reasonable request.
